# Transient musical hallucinations in a young adult male associated with alcohol withdrawal

**DOI:** 10.1192/bjo.2021.710

**Published:** 2021-06-18

**Authors:** Mao Lim, Graham Blackman, Anthony David, Fahmida Mannan

**Affiliations:** 1 Cambridge University Hospitals NHS Foundation Trust; 2 Department of Psychosis Studies, Institute of Psychiatry, Psychology and Neuroscience, King's College London; 3 Institute of Mental Health, University College London

## Abstract

**Aims:**

We present the case of a 25-year-old male who presented to A&E with isolated musical hallucinations, in the absence of audiological or neurological disease.

**Background:**

Musical hallucinations (MH) are a form of complex auditory hallucinations whereby an individual experiences an instrumental and/or vocal melody in the absence of auditory stimuli.

**Result:**

The patient had a history of recreational drug use and a family history of psychosis. Hallucinations, which were preceded by discontinuation of alcohol and re-initiation of citalopram for depression, resolved spontaneously after three days.

**Conclusion:**

Aetiological factors are discussed alongside the existing literature. Whilst the underlying mechanisms underpinning musical hallucinations remains elusive, the case illustrates the potential role of alcohol withdrawal, serotonin toxicity, recreational drug use and genetic vulnerability.

